# A Designer Synbiotic Attenuates Chronic-Binge Ethanol-Induced Gut-Liver Injury in Mice

**DOI:** 10.3390/nu11010097

**Published:** 2019-01-05

**Authors:** Sanjoy Roychowdhury, Bryan Glueck, Yingchun Han, Mahmoud Ali Mohammad, Gail A. M. Cresci

**Affiliations:** 1 Department of Inflammation and Immunity, Lerner Research Institute, Cleveland Clinic, Cleveland, OH 44195, USA; roychos@ccf.org (S.R.); glueckb@ccf.org (B.G.); hany2@ccf.org (Y.H.); 2 Department of Pediatrics, Children’s Nutrition Research Center, U.S. Department of Agriculture/Agricultural Research Service, Baylor College of Medicine, Houston, TX 77030, USA; Mohammad@bcm.edu; 3 Department of Pediatric Gastroenterology, Cleveland Clinic Children’s Hospital, Cleveland, OH 44195, USA; 4 Center for Human Nutrition, Digestive Disease Institute, Cleveland Clinic, Cleveland, OH 44195, USA

**Keywords:** short chain fatty acids, butyrate, propionate, synbiotic, intestine, liver, inflammation, steatosis, ethanol, tight junction proteins

## Abstract

Gut dysbiosis and altered short-chain fatty acids are associated with ethanol-induced liver injury. SCFA are fermentation byproducts of the gut microbiota known to have many beneficial biological effects. We tested if a designer synbiotic could protect against ethanol-induced gut-liver injury. C57BL/6 female mice were exposed to chronic-binge ethanol feeding consisting of ethanol (5% vol/vol) for 10 days, followed by a single gavage (5 g/kg body weight) 6 h before euthanasia. A group of mice also received oral supplementation daily with a designer synbiotic, and another group received fecal slurry (FS); control animals received saline. Control mice were isocalorically substituted maltose dextran for ethanol over the entire exposure period. Ethanol exposure reduced expression of tight junction proteins in the proximal colon and induced hepatocyte injury and steatosis. Synbiotic supplementation not only mitigated losses in tight junction protein expression, but also prevented ethanol-induced steatosis and hepatocyte injury. Ethanol exposure also increased hepatic inflammation and oxidative stress, which was also attenuated by synbiotic supplementation. Mice receiving FS were not protected from ethanol-induced liver injury or steatosis. Results were associated with luminal SCFA levels and SCFA transporter expression in the proximal colon and liver. These results indicate supplementation with a designer synbiotic is effective in attenuating chronic-binge ethanol-induced gut-liver injury and steatosis in mice, and highlight the beneficial effects of the gut microbial fermentation byproducts.

## 1. Introduction

Excessive alcohol consumption can lead to alcoholic liver disease (ALD), liver manifestations that may progress through a series of pathologies starting with fatty liver, or hepatic steatosis. In a subset of people, hepatic steatosis can proceed to steatohepatitis, characterized by inflammation and hepatic cell death. Steatohepatitis can further proceed to fibrosis, eventually leading to cirrhosis, which encompasses massive hepatocellular injury [1]. 

Following ingestion, ethanol enters the liver from the intestine through the portal circulation. Expressing high levels of two enzymes which oxidize ethanol, alcohol dehydrogenase and cytochrome P 450 2E1 (CYP2E1), the liver is the primary site of ethanol metabolism [2]. Ethanol oxidation leads to the depletion of antioxidants and formation of reactive oxygen species (ROS), which are generated in association with the mitochondrial electron transport system, CYP2E1, and by activated Kupffer cells, liver resident macrophages [1].

High-dose chronic ethanol consumption is now known to disrupt gut microbial concentration and diversity, otherwise known as gut dysbiosis. This resulting microbial disturbance allows for the overgrowth of opportunistic pathogens, which promotes gut barrier disruption and release of endotoxins into circulation [3]. Endotoxins including lipopolysaccharides (LPSs) can enter the liver through portal circulation and activate pattern recognition receptors including toll-like receptors [4]. Mice deficient in toll-like receptor-4 are protected from ethanol-induced liver injury [5]. 

In a mouse model of chronic ethanol exposure, gut microbial shifts occurred that were characterized by reduced *Bacteroidetes* and *Firmicutes* and expanded *Actinobacteria* and *Proteobacteria* phyla. Overgrowth of gram negative bacteria and depletion of butyrate-producing bacteria is associated with increased endotoxemia, hepatic inflammation, steatosis, and gut permeability [6]. Ethanol-induced shifts in gut microbial populations are associated with altered microbial fermentation byproducts such as the short-chain fatty acids (SCFA), acetate, propionate, and butyrate [7]. Butyrate is important for maintaining gut health, and plays a protective role in various pathologies including toxin-induced acute liver failure [8] and ischemia-reperfusion injury [9]. Tributyrin, a butyrate pro-drug, protects against ethanol-induced gut-liver injury and hepatic inflammation in mice [10,11]. 

Probiotics, defined as “live microorganisms which when administered in adequate amounts confer a health benefit on the host”, are frequently sourced from a healthy gut microbiome [12]. Clinical trials have shown that probiotics are beneficial in treating patients with ALD [5,13]. A recent study demonstrated that supplementation with the probiotic *Lactobacillus fermentum* was beneficial against ethanol-induced liver injury [14]. Probiotics containing butyrate-producing bacteria have been shown to play protective roles in multiple disease models including Crohn’s disease [15] and non-alcoholic fatty liver disease [16]. 

The beneficial effects of probiotic supplementation is both strain-specific and transient, therefore may not result in consistent long-term gut microbial colonization changes. Since ethanol exposure alters the gut microbiota, providing only a probiotic may not be sufficient to fully restore microbial homeostasis or generate beneficial fermentation byproducts. Prebiotics are defined as “selectively fermented ingredients that result in specific changes in the composition and/or activity of the gastrointestinal microbiota, thus conferring benefit(s) upon host health” [17]. Often ethanol consumption and gut dysbiosis is associated with suboptimal nutritional intake, therefore the ideal metabolic substrate for the probiotic bacteria, fermentable soluble fiber, may not be available. Therefore, targeting the gut microbiota with both a probiotic and prebiotic simultaneously (synbiotic) can overcome this common situation. It would be ideal to design a synbiotic for a particular target such as to generate a metabolic byproduct. Due to the demonstrated beneficial effects of butyrate on ethanol-induced gut-liver injury, synbiotic supplementation comprised of a butyrate-producing bacteria and a prebiotic known to yield butyrate upon fermentation could be an effective preventative strategy for ALD. 

This study aimed to evaluate the effects of supplementing a novel designer synbiotic, comprised of a butyrate-producing bacteria (*Faecalibacterium prausnitzii*) and a butyrate-yielding prebiotic (potato starch), on mitigating chronic-binge ethanol-induced gut-liver injury and inflammation in mice. As an alternative, this study also tested the effect of fecal slurry supplementation with the premise that healthy stool is the “ultimate” synbiotic as it contains trillions of commensal microbes and their fermentation byproducts. The present study provides novel findings on the preventative effects of designer synbiotic supplementation in mitigating chronic-binge ethanol-induced gut-liver injury and hepatic steatosis by altering SCFA and their transporters in the proximal colon and liver. 

## 2. Materials and Methods 

### 2.1. Materials 

Female C57BL/6J mice (10–12 weeks old) were purchased from Jackson Labs (Bar Harbor, ME, USA). Lieber-DeCarli ethanol and control diets were purchased from Dyets (Bethlehem, PA, USA). *Faecalibacterium prausnitzii* 27766 was purchased from ATCC (Manassas, VA, USA); potato starch was purchased from Sigma-Aldrich (St. Louis, MO, USA). Antibodies were from the following sources: Monocarboxylate transporter-1 (MCT1), SLC5A8, and zonula occludens-1 (ZO1) from Thermo Fisher (Rockford, IL, USA); CYP2E1 and NHE3 from Abcam (Eugene, OR, USA); HSC70 from Santa Cruz Biotechnology Inc. (Santa Cruz, CA, USA); TNFα from Fitzgerald (Acton, MA, USA); 4-hydroxynonenal from Alpha Diagnostics (San Antonio, TX, USA); and Claudin-3, Alexa Fluor 488 or 568 tagged IgGs from Invitrogen (Carlsbad, CA, USA). 

### 2.2. Ethanol Exposure Model and Supplementations

All procedures using animals were approved by the Cleveland Clinic Institutional Animal Care and Use Committee. Mice were housed in cages (2 animals/cage) with microisolator lids. Mice were randomized into ethanol-fed and pair-fed groups and then adapted to a control liquid diet for five days. The ethanol-fed group was allowed free access to a diet containing 5% (vol/vol) ethanol; control mice were those pair-fed diets containing isocalorically substituted maltose dextrins for ethanol over the entire exposure period. On day 11, mice were orally gavaged with an ethanol bolus (5 g/kg body weight) and euthanized 6 h post-gavage. During ethanol exposure, a group of mice were provided with a synbiotic that consisted of *Faecalibacterium prausnitzii* (FP: 6 _log10_ CFU/10 μL daily) and potato starch (PS: 20% w/v, 20 μL daily) by oral gavage; control group mice were gavaged with 0.9% normal saline. The concentration/dose of the synbiotic were based off of prior studies demonstrating beneficial effects [18]. In a separate trial, mice received a fecal slurry (FS) instead of the synbiotic. FS consisted of the collection of mouse fecal pellets prior to the beginning of the pair-fed diet, which was stored at −80 °C. Fecal pellets were then re-suspended in 0.9% normal saline and the FS was gavaged daily; control mice received 0.9% normal saline. Blood samples were collected into non-heparinized syringes from the posterior vena cava, livers were blanched with saline via the portal vein and then excised. Portions of each liver were then either fixed in formalin or frozen in optimal cutting temperature (OCT) compound (Sakura Finetek U.S.A., Inc., Torrance, CA, USA) for histology, frozen in RNAlater (Qiagen, Valencia, CA, USA), or flash frozen in liquid nitrogen and stored at −80 °C until further analysis. Blood was transferred to EDTA-containing tubes, plasma was isolated and stored at −80 °C until further use.

### 2.3. Biochemical Assays

Total liver triglycerides were measured using the Triglyceride Reagent Kit from Pointe Scientific (Lincoln Park, MI, USA). Plasma samples, collected as detailed above, were assayed for alanine aminotransferase (ALT) using a commercially available enzymatic assay kit (Diagnostic Chemicals, Oxford, CT, USA) as per the manufacturer’s protocol. 

### 2.4. Immunohistochemistry

Paraffin-embedded slides were de-paraffinized and underwent antigen retrieval by treating in Tris-EDTA buffer (pH 8) at 95 °C for 20 min, followed by 20 min exposure on the bench top. Following antigen retrieval, slides were washed three times with phosphate buffered saline (PBS), blocked with 10% normal goat serum or 2% bovine serum albumin and then incubated with the primary antibodies diluted in blocking buffer overnight at 4 °C. Proximal colon sections were stained for zonula occludens-1 (ZO1), Claudin-3, solute carrier family 5 member 8 (SLC5A8), monocarboxylate transporter isoform 1 (MCT1), and sodium-hydrogen exchanger-3 (NHE3) in the proximal colon; and in the liver stained for SLC5A8, tumor necrosis factor-α (TNFα), and 4-hydroxynonenal (4-HNE). The next day, slides were washed with PBS three times and incubated with the respective secondary antibodies tagged with Alexa Fluor 488 or 568 or horseradish-peroxidase conjugated IgG in the dark for 1 h. Slides were then washed three times with PBS and mounted with DAPI-containing mounting media (Vectashield H-1200, Vector Laboratories, Burlingame, CA, USA). For bright-field microscopy, at the end of the incubation with the primary antibody, sections were conjugated using Vectastain Elite Rabbit IgG Kit (Vector Labs) and visualized using 3,3 diaminobenzidine (DAB) or the 3-amino-9-ethylcarbazole (AEC)-substrate chromogen. Following development, slides were then counterstained using Gill’s hematoxylin (Vector Labs). Images were acquired using a bright field microscope or an upright confocal microscope (Leica Microsystems, Buffalo Grove, IL, USA) for fluorescent images. All of the parameters in the microscope were kept constant for all images of a specific protein. No specific immunostaining was seen in sections incubated with PBS rather than the primary antibody. Slides were coded and at least three images were acquired per tissue section, with at least eight mice per experimental group. Images were semi-quantified using Image Pro Plus software (Media Cybernetics, Bethesda, MD, USA). A color intensity was manually selected as an indicator of the positive staining and a color file was created with specific hue, saturation, and intensity. This color file, designating a specific range of the signal, was applied to all images to measure the positive expression of that protein and the results were exported to an Excel spreadsheet. The sum of the positive area, obtained from the software-driven analysis, was considered as an indicator of the expression of that specific protein. 

### 2.5. Oil Red O Staining

Liver sections were dried in air for 10 min followed by staining with a fresh Oil Red O solution (Sigma, St. Louis, MO, USA) for 10 min, then rinsed in water, and counterstained with hematoxylin. The quantification of slides was the same as that described in the immunohistochemistry section above. 

### 2.6. Western Blot

Liver homogenates were prepared and protein concentrations were determined for immunoblotting as follows: Liver Homogenization: Frozen liver tissue was homogenized in lysis buffer (1% Triton X-100, 50 mM Tris-HCl pH 7.4, 150 mM NaCl, 1 mM EDTA pH 8, 0.1% Na-deoxycholate, 0.1% SDS) containing Pierce Protease and Phosphatase Inhibitor Mini Tablets (Thermo Fisher, Waltham, MA, USA) as recommended by the manufacturer. Samples were centrifuged at 16,000× *g* for 15 min at 4 °C and the supernatant was collected. Protein concentrations were measured using the DC Protein Assay from Bio-Rad (Hercules, CA, USA). Samples were then normalized and prepared in Laemmli buffer and boiled for 5 min. Protein (30 µg/20 µL) was resolved on 10% polyacrylamide gels and transferred to polyvinylidene fluoride membranes. Membranes were probed with antibodies specific for CYP2E1, NHE3, MCT1, and SLC5A8; HSC70 was used as the loading control. At least six to eight mice per treatment group were analyzed. Immuno-reactive protein expression was detected using enhanced chemiluminescence and signal intensities were determined by densitometry using ImageJ software (NIH, Bethesda, MD, USA). In brief, gel images were converted to grayscale images, a rectangular box was drawn over the protein band to specify the protein of interest, and the intensity was measured. The same size rectangular box was then moved to subsequent lanes at the same molecular weight level and intensities were sequentially measured. The intensity of the protein of interest from a particular sample was then normalized to the intensity of the loading control protein (HSC70) or the same sample. 

### 2.7. Real Time Polymerase Chain Reaction

Total RNA was isolated from the proximal colon samples. As previously described, 2 µg of total RNA was reverse transcribed [18]. A QuantStudio 5 analyzer (Applied Biosystems, Foster City, CA, USA) was used to perform real-time PCR amplification with PowerSYBR qRT-PCR kits (Applied Biosystems) for primers: sodium-hydrogen exchanger-3 (NHE3; FWD: CACCTTCAAATGGCACCACG/REV: TGTGGGACAGGTGAAAGACG) and glyceraldehyde 3-phosphate dehydrogenase (GAPDH: FWD: AGGTCGGTGTGAACGGATTTG/REV: TGTAGACCATGTAGTTGAGGTCA). The comparative threshold (Ct) method was used to determine the relative amount of target mRNA to the values of the housekeeping gene, GAPDH. Graphs are represented as fold change relative to saline treated pair-fed mice.

### 2.8. Detection of Short-Chain Fatty Acids in Fecal Samples

Fecal short chain fatty acids (SCFA) were measured using gas chromatography/mass spectrometry (GCMS) after derivatization to the corresponding pentaflurobenzyl (PFBBr) derivative according to a previously established method [19] with modifications. Fatty acid (FA) standards (acetic, propionic, butyric, and isoutyric) and lactic and pyruvic acids were obtained from Sigma-Aldrich Inc. (St. Louis, MO, USA). The uniformly deuterium labeled FAs (atom 99%) (acetic, propionic and butyric) and ^13^C_3_-lactate were obtained from Cambridge Isotope Laboratory (Andover, MA, USA) and used as internal standards. Acetone, hexane, dichloromethane, ethanol, and all solvents were HPLC grade. *O*-(2, 3, 4, 5, 6-pentafluorbenzyl) bromide (PFB)-Br and tetrabutylammonium hydrogen sulfate (TBA) were obtained from Aldrich Chemicals Co. Inc. (Milwaukee, WI, USA). Briefly, about 20–30 mg of fecal sample was accurately weighed into a clean 2 mL Eppendorf vial to which, 0.5 mL solution containing the labeled FAs internal standards (100 nmoles d4-acetate, 50 nmoles d6-propionate, 25 nmoles d8-butyrate, and 5 nmoles d10-valerate) and 25 nmoles ^13^C3-lactate was added and vortexed for 10 min. The samples were then centrifuged at 10,000 rpm for 10 min. Aliquots of 200 μL of the supernatant were aspirated for derivatization with PFBr by transferring into a 4 mL Teflon-lined screw cap vial and adding 250 μL of 0.1 M TBA counter ion solution and vortexed for 5 min. An aliquot of 400 μL of 0.13 M PFB-Br in dichloromethane was added to each tube and vortexed vigorously for 10 min. The tubes were kept at room temperature overnight to complete the derivatization reaction. On the following morning, 1 mL of hexane (containing 10% ethanol) was added, and tubes were vortexed for 5 min and were subsequently centrifuged for 15 min at 3000 rpm at 4 °C. The supernatant (organic layer), which contains the PFB-FA esters, was transferred to a clean GCMS vial and injected to the GCMS. A set of FA external standards covering the range of SCFA concentrations in the fecal samples were prepared (including adding internal standards), derivatized, and run simultaneously with the samples. The derivatized samples and standards were analyzed using a Hewlett Packard GCMS (GC 6890; MS 5973) with an Rtx^®^-225 column (30 m × 0.25 mm × 0.25 μM, Restek Corporation, Bellefonte, PA, USA). The conditions for the GC were as follows: Injector: 250 °C (splitless injection of samples); oven: 60 °C for 1.0 min; ramp, 15 °C/min to 240 °C; hold at 240 °C for 10 min. Methane negative chemical ionization analyses were performed as the reagent gas. Data were acquired in selective ion monitoring (SIM) mode. Mass fragments of different FA-PFBBr esters used in quantification have been previously reported [19]. Peak areas of the analyte or of the standards were measured, and the ratio of the area from the analyte-derived ion to that from the internal standard was calculated. The ratios were then compared with the calibration curves for the analyte prepared from the standards to determine the concentration of individual SCFAs. The concentrations of the short chain fatty acids are expressed as µmol/animal or as a percent of the sum of all three major short chain fatty acids including acetate, propionate, and butyrate. All fecal fatty acids quantification measurements were made in the Stable Isotope Core Laboratory of the Children’s Nutrition Research Center.

### 2.9. Statistical Analysis

All data were expressed as the mean ± standard error of the mean (SEM) with *n* = 8–16 mice per treatment groups. A Student *t*-test was used for the parametric analysis of two groups; analysis of variance was used for a comparison of multiple groups with a Tukey’s post hoc test for multiple comparisons. Data were log-transformed to obtain a normal distribution as needed. Statistical significance was defined as *p* < 0.05. The analysis was performed using Prism software Version 5.02 (GraphPad Software, San Diego, CA, USA).

## 3. Results

### 3.1. Synbiotic Supplementation Altered SCFA Levels in Cecum

**Figure 1:**
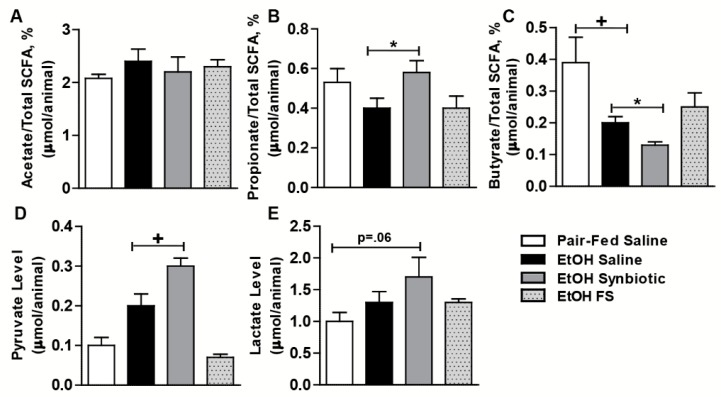
Short-chain fatty acid profiles in mouse cecum. Mice were fed an ethanol (5% v/v) containing liquid diet or pair-fed a diet with maltose dextrin isocalorically substituted for ethanol for 10 days. Mice were orally supplemented daily with butyrate-targeting synbiotic, fecal slurry, or saline. Mice were then treated with a single 5 g/kg gavage of ethanol the next day. At 6 h post gavage, cecum was collected and flash frozen and stored at −80 °C until analyzed by GC-MS for SCFA. (**A**) Acetate %; (**B**) propionate %; (**C**) butyrate %; (**D**) pyruvate; (**E**) lactate. Values are as a percentage of total SCFA (acetate, propionate, butyrate) or as mean concentration (µmol/animal cecum content) ± SEM. * *p* = 0.03; **+** *p* = 0.01. Data are representative of at least 8 to 12 mice per treatment group.

Daily oral supplementation with the synbiotic was well-tolerated in mice. We analyzed the luminal content from the cecum of mice for acetate, propionate, butyrate, pyruvate, and lactate via GC/MS and found that the levels varied between groups. While there was a trend towards an increased percentage of acetate in the ethanol-saline treated animals, this group had lower amounts of propionate, pyruvate, and lactate compared to the ethanol treated animals supplemented with the synbiotic (Figure 1). Surprisingly, butyrate was higher in the ethanol-saline when compared to the ethanol-synbiotic mice (Figure 1C). 

### 3.2. Synbiotic Supplementation Mitigated Losses in SCFA Transport Mechanisms Altered by Ethanol Exposure

**Figure 2:**
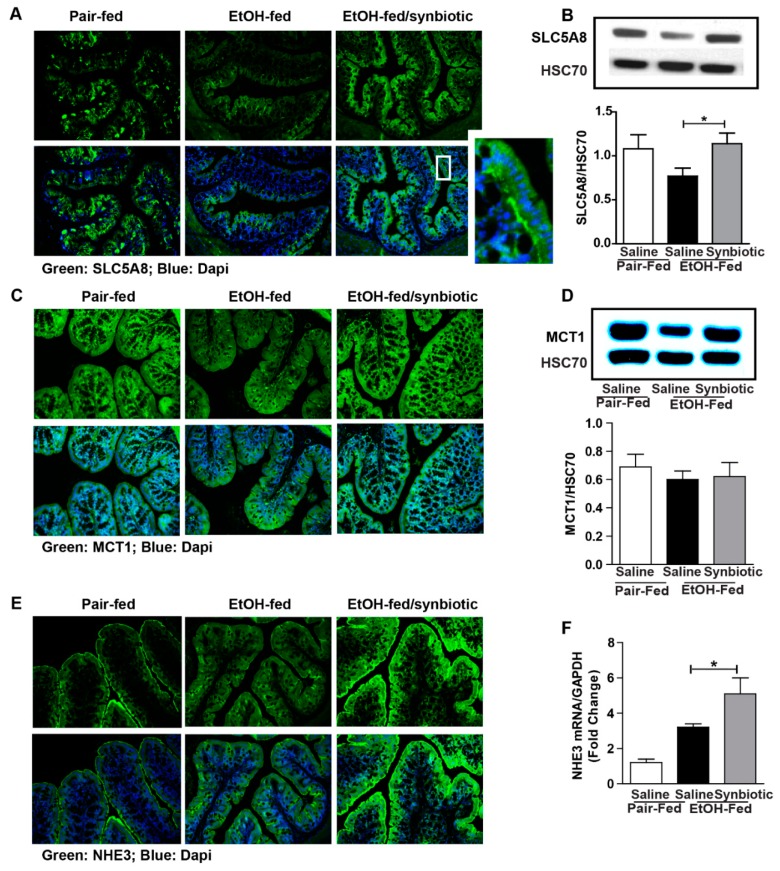
Short-chain fatty acid transporter and anion exchanger expression in the proximal colon. Mice were treated as described in Figure 1. Proximal colon was collected and whole tissue was flash frozen for immunoblotting, fixed in formalin and paraffin embedded for histology, or RNA was prepared and used for qRT-PCR analysis. (**A**) SLC5A8 (green) was visualized by immunohistochemistry and (**B**) immunoblot of SLC5A8 using HSC70 as internal control; (**C**) MCT1 (green) was visualized by immunohistochemistry and (**D**) immunoblot of MCT1 using HSC70 as internal control; (**E**) NHE3 (green) was visualized by immunohistochemistry; (**F**) NHE3 mRNA expressed in proximal colon presented as fold change. A selected area was cropped and enlarged. All images were acquired using a 20× objective. Images are representative of at least replicate images captured per mouse in 8 to 12 mice per treatment group. Band densities were analyzed using ImageJ software and normalized to HSC70. Values represent means ± SEM. * *p* < 0.05.

Butyrate is important for intestinal homeostasis and cellular energy. Although butyrate is the least abundant of the three predominant SCFAs (acetate ~60%, propionate 25%, and butyrate 15%), it is the major fuel source for the colonocyte [20]. Butyrate can be absorbed across the colonic apical membrane by diffusion of the undissociated form, and by active transport of the dissociated form via SCFA transporters. SLC5A8 is a sodium-coupled co-transporter, and MCT1 is functionally coupled to a transmembrane H^+^ gradient, NHE3 [20]. Butyrate transporters are most abundant in the large intestine, and more physiologically active in the proximal colon due to a higher concentration of butyrate and lower luminal pH in this region [21]. Due to the variability in butyrate between treatment groups, we assessed the potential for absorbing butyrate by evaluating the protein expression of SLC5A8, MCT1, and NHE3 in the proximal colon. While the expression of MCT1 was not different between treatment groups (Figure 2C,D), mice exposed to chronic-binge ethanol had visibly less immunostaining for SLC5A8 and NHE3 (Figure 2A,D), and less gene expression for NHE3 (Figure 2F) when compared to the synbiotic supplemented mice exposed to ethanol. Diminished expression of both SLC5A8 and NHE3 could attribute to less butyrate being transported from the gut lumen, and thus explain the higher butyrate in the cecum of mice in the ethanol-saline group (Figure 1C). 

**Figure 3:**
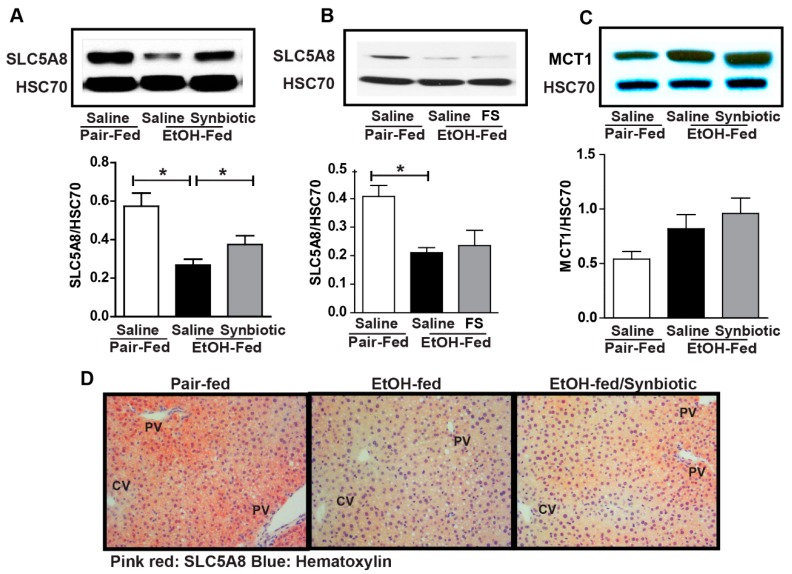
Short-chain fatty acid transporter expression in the liver. Mice were treated as described in Figure 1. Liver was collected and flash frozen for immunoblotting or fixed in formalin and paraffin embedded for histology. (**A**,**B**) immunoblot of SLC5A8 using HSC70 as the internal control; (**C**) immunoblot of MCT1 using HSC70 as the internal control; (**D**) SLC5A8 (pink-red) was visualized by immunohistochemistry. All images were acquired using a 20× objective. Images are representative of at least replicate images captured per mouse in 8 to 12 mice per treatment group. Band densities were analyzed using ImageJ software and normalized to HSC70. Values represent means ± SEM. * *p* < 0.05.

While the majority of the absorbed butyrate is oxidized in the colonocyte, small amounts can be absorbed and then enter the liver via the portal vein [22]. Therefore, we assessed the expression of SCFA transporters in the liver. Ethanol exposure had a differential effect on MCT1 and SLC5A8. MCT1 was induced by ethanol while SLC5A8 was reduced (Figure 3). Mice supplemented with the synbiotic had higher SLC5A8 expression by Western blot and immunohistochemistry, and a trend towards higher MCT1 expression (Figure 3A,C,D). SLC5A8 expression appeared to predominate around the portal vein in hepatocytes, the location of butyrate entry into the liver (Figure 3D). Interestingly, oral supplementation with healthy fecal slurry did not affect the ethanol downregulation of SLC5A8 (Figure 3B).

### 3.3. Ethanol-Induced Alterations of Tight Junction Proteins in Proximal Colon Was Mitigated with Synbiotic Supplementation

**Figure 4:**
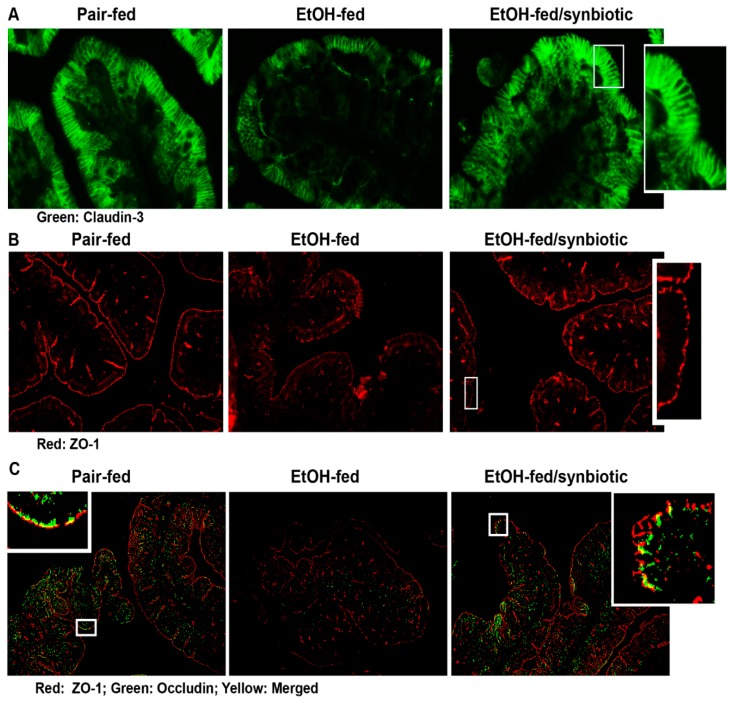
Tight junction protein expression in the proximal colon. Mice were treated as described in Figure 1. The proximal colon was collected and fixed in formalin and paraffin embedded for histology. (**A**) claudin-3 (green); (**B**) ZO-1 (red); and (**C**) ZO-1 (red) and occludin (green) and merged ZO-1/occludin (yellow) were visualized by immunohistochemistry. A selected area was cropped and enlarged. All images were acquired using a 20× objective. Images are representative of at least replicate images captured per mouse in 8 to 12 mice per treatment group.

The intestinal epithelial barrier is composed of apical intercellular junctional proteins known as tight junctions (TJ) and associated proteins known as the adherens junctions. The TJ are composed of transmembrane proteins (e.g., claudins), integral membrane proteins (e.g., occludin), junction adhesion molecules, and cytoplasmic zona occludens (ZO) proteins (e.g., ZO-1, -2 and-3) which connect the TJ complex intracellularly with the actin cytoskeleton [23]. Disruption in these proteins may allow for the paracellular transport of endotoxin from the gut lumen to the liver via the portal vein. Ethanol is known to negatively impact the intestinal barrier [24]. Butyrate has a protective effect on the intestinal epithelial barrier in both mitigating its disruption and assisting in its repair [20]. Our group has previously reported that supplementation with tributyrin, a prodrug of butyrate, preserves tight junction protein expression in the mouse ileum and proximal colon during both chronic and acute ethanol exposure [10,11]. Therefore, we investigated whether synbiotic supplementation had an effect on tight junction proteins during chronic-binge ethanol exposure. As expected, chronic-binge ethanol exposure diminished the expression of tight junction proteins, claudin-3, ZO-1, and ZO-1/occludin co-localization in the proximal colon (Figure 4A–C). Co-supplementation with synbiotic preserved immunoreactive staining intensity to similar patterns visualized in the pair-fed control mice (Figure 4A,B). Synbiotic supplementation also preserved the co-localization of ZO-1 and occludin, which was visibly disrupted in the ethanol-saline treated mice (Figure 4C).

### 3.4. Synbiotic Supplementation Attenuated against Chronic-Binge Ethanol-Induced Steatosis and Liver Injury

**Figure 5:**
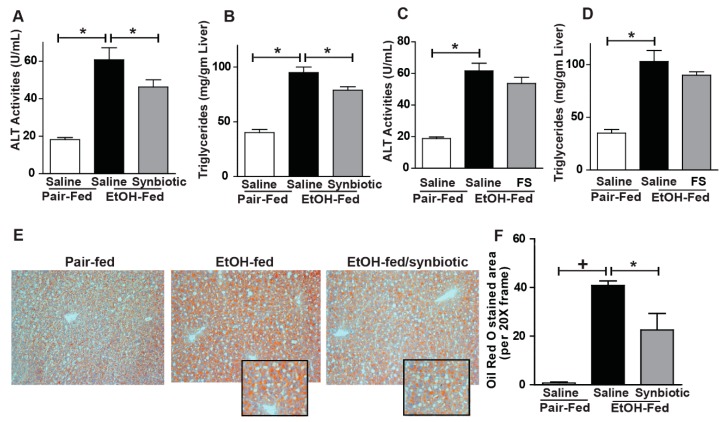
Liver injury and steatosis. Mice were treated as described in Figure 1. (**A**,**C**) ALT activity was measured in plasma; (**B**,**D**) Hepatic triglyceride content was measured in whole liver homogenate; (**E**) OCT-embedded frozen liver sections were stained with Oil-Red O to visualize neutral lipids. Inset shows cropped images. (**F**) Images are representative of 8–12 mice per treatment group and were acquired using a 20× objective and semi-quantified using Image Pro Plus software. Values represent means ± SEM. * *p* < 0.05; **+** *p* < 0.01.

Disruption of the intestinal barrier during ethanol exposure is associated with liver injury. Since we found that chronic-binge ethanol exposure impaired intestinal barrier integrity, we investigated whether synbiotic supplementation would also modify liver injury and steatosis. Indeed, synbiotic supplementation attenuated chronic-binge ethanol-induced increases in plasma ALT and hepatic triglyceride accumulation (Figure 5A,B). Staining neutral lipids with Oil Red O also confirmed that synbiotic supplementation reduced chronic-binge ethanol-induced lipid accumulation in the mouse liver (Figure 5E,F). Supplementation with just fecal slurry did not affect liver injury or steatosis induced by chronic-binge ethanol exposure (Figure 5C,D).

### 3.5. Synbiotic Supplementation Reduces Chronic-Binge Ethanol-Induced TNFα Expression and 4-HNE-Adduct Accumulation in Mouse Liver

**Figure 6:**
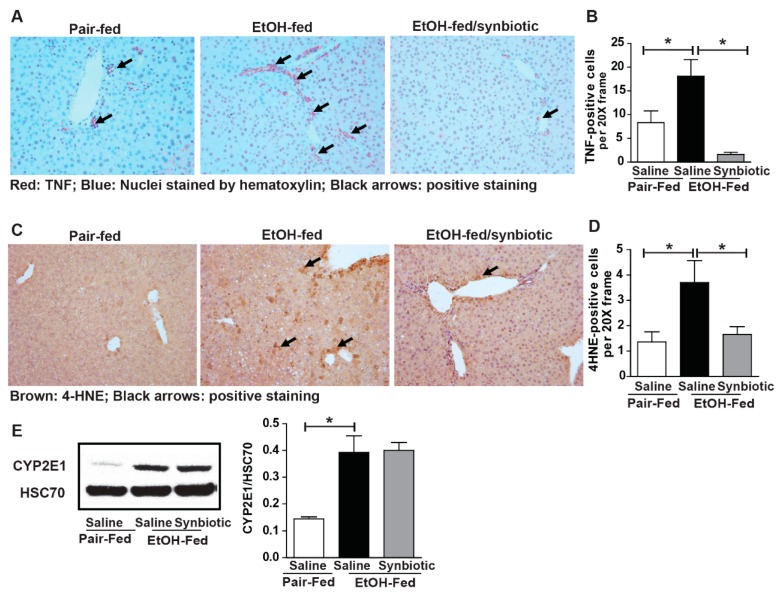
Hepatic TNFα expression and oxidative stress. Mice were treated as described in Figure 1. Paraffin-embedded livers were subjected to immunostaining for TNFα (**A**,**B**) and 4-hydroxynonenal (4-HNE) (**C**,**D**). Images are representative of 8–12 mice per treatment group and were acquired using a 20× objective, and positive staining was quantified using Image-Pro Plus software and analyzed. Black arrows (**A**,**C**) indicate positive staining. Expression of CYP2E1 (**E**) in the liver was detected by immunoblot analysis using HSC70 as a loading control. Band densities were analyzed using ImageJ software and normalized to HSC70. Values represent means ± SEM. * *p* < 0.05.

Ethanol metabolism primarily occurs in the liver and induces oxidative stress and inflammation [25]. Increased expression of TNFα is implicated in the induction of hepatocyte death following ethanol exposure [26]. Many studies have shown butyrate to have antioxidant and anti-inflammatory properties [20]. Since we found chronic-binge ethanol exposure impaired hepatic expression of SCFA transporters, we hypothesized that opposed to saline, synbiotic supplementation would attenuate ethanol-induced oxidative stress and hepatic inflammatory mediators. Ethanol exposure increased expression of TNFα (Figure 6A,B), an inflammatory mediator, and accumulation of 4-HNE adducts, a dosimeter of oxidative stress, around the portal veins in the liver (Figure 6C,D). Synbiotic supplementation reduced induction of TNFα expression and 4-HNE-adduct formation in mouse liver (Figure 6A–D). We evaluated if synbiotic-mediated reduction of ethanol-induced oxidative stress was an outcome of altered ethanol metabolism. We found that the expression of CYP2E1, the major ethanol metabolizing enzyme, was induced equally following ethanol exposure in animals receiving both saline and synbiotic supplementation, demonstrating that synbiotic-mediated protection against oxidative stress is independent of CYP2E1 (Figure 6E).

## 4. Discussion

As growing evidence associates ethanol-induced pathology with gut dysbiosis, implementing strategies which target consequential gut microbial and metabolic byproduct disruption is logical, particularly since there is a lack of effective treatment options for ALD. In the present study, we found chronic-binge ethanol exposure negatively impacted gut-liver homeostasis, decreasing expression of tight junction proteins in the proximal colon as well as inducing liver injury, inflammation, oxidative stress, and hepatic steatosis. Here for the first time, we provide evidence of a beneficial effect of prophylactic supplementation with a designer synbiotic in mitigating these negative insults in the proximal colon and liver of mice. 

Supplementation with probiotics and synbiotics is evolving as new therapeutic approaches to treat hepato-pathologies including diet-induced obesity and metabolic syndrome [27]. Synbiotic supplements comprised of fermentable fibers assist in the survival and/or growth of specific beneficial bacterial populations in the gut, and serve as important precursors for intra-luminal generation of SCFAs in the colon [28,29]. Acetate, propionate, and butyrate are the most abundant SCFA representing 90–95% of SCFA in the colon [30]. While probiotic mechanisms are strain specific, most probiotic and synbiotic studies are not designed to target specific metabolic byproducts. Since ethanol is known to deplete both luminal butyrate-producing bacteria and butyrate [7], and our prior work has demonstrated the beneficial effects of butyrate supplementation during ethanol exposure [10,11], here we tested the efficacy of a synbiotic deliberately designed to target SCFA in the gut lumen and counteract inflammation. In order to facilitate this, we combined a butyrate-producing commensal bacteria, *F. prausnitzii*, with a butyrate-yielding prebiotic, potato starch [31]. A dominant member of the *Clostridium leptum* subgroup, *F. prausnitzii* is considered an important gut microbe of a healthy gut microbiota, comprising >5% of the total gut microbiota in healthy humans [32]. Due to its ability to generate butyrate, which favorably modulates the immune system and oxidative stress [33], *F. prausnitzii* is also known to possess anti-inflammatory properties [34], and improve high-fat diet induced liver injury and adipose tissue inflammation in mice [35].

Butyrate can be directly generated via two different pathways in the gut. The less common route is the butyrate kinase pathway, which employs phosphotransbutyrylase and butyrate kinase enzymes to convert butyryl-Co-A into butyrate. The butyryl-CoA:acetate CoA-transferase pathway is used by the majority of gut butyrate-producers including *Faecalibacterium* [36]. Additionally, bacterial cross-feeding largely impacts the final SCFA balance. Cross-feeding mechanisms consist either in metabolic cross-feeding, the utilization of end products from the metabolism of a given microorganism by one another, and/or substrate cross-feeding, which is the utilization of one microorganism of the generated SCFA formed by another one [37]. Utilizing stable isotope tracers, Den Besten et al. demonstrated that bacterial cross-feeding occurred between acetate to butyrate, at a lower extent between butyrate to propionate, and was nearly absent between acetate and propionate [38]. Utilization of acetate by *F. prausnitzii* and *Roseburia* sp. has been shown in vitro [39]. Additionally, some butyrate and propionate-producing bacteria are able to use lactate, and may employ this to avoid metabolic acidosis [40]. Acetate is the final metabolic end product in ethanol metabolism. Interestingly, our data corroborated others showing ethanol-saline mice had higher acetate with lower ratios of propionate, pyruvate, and lactate than synbiotic supplemented mice [7]. Additionally, mice supplemented with fecal slurry exhibited similar trends in SCFA ratios to those treated with saline. This suggests that supplemented *F. prausnitzii* could have assisted in the cross-feeding of acetate or lactate into the other SCFAs such as propionate in this study.

Surprisingly, the butyrate/total SCFA ratio in the cecum was lower in the synbiotic supplemented mice exposed to ethanol than those receiving saline. Butyrate is known to be the primary fuel source for the colonocyte, with ~70% of generated butyrate being metabolized by colonic epithelium [22]. Luminal butyrate can be absorbed by passive diffusion or active transport into the colonocyte. SLC5A8 is a sodium-coupled active transporter highly expressed in the cecum and colon and its expression is diminished in situations of gut dysbiosis, while transporter expression is protected with supplementation with probiotic or tributyrin [11,41,42]. There are multiple isoforms with variable tissue expression for MCTs [42]. MCT1 is a H^+^-coupled co-transporter, coupled with NHE3, for monocarboxylates and SCFA and is expressed in the apical and basolateral membrane of the colon and in the liver [42,43]. Butyrate has been shown to stimulate promoter activity and the expression of NHE3 in colonic epithelial cells in vitro [18]. Decreased butyrate uptake by intestinal epithelial cells occurs with an increased pH [44]. Lower expression of NHE3 would result in an elevated pH due to decreased Na^+^-H^+^ exchange across the apical membrane. Thus, decreased expression of the butyrate transporters and Na^+^/H^+^ exchanger would suggest defective entry of butyrate into colonic epithelial cells. Ethanol and its immediate metabolite acetaldehyde as well as oxidative stress, which is induced by ethanol, also reduce butyrate uptake as demonstrated in vitro [44,45]. We showed a decreased expression of SLC5A8 and NHE3 in the proximal colon in the ethanol-saline treated animals, suggesting that the higher luminal butyrate found in these animals may be a result of decreased transport into the colonocyte. Butyrate is known to support intestinal tight junction proteins, therefore lower luminal butyrate levels and a higher transporter expression coincides with the protective effect of synbiotic supplementation during ethanol exposure on the colonic expression of tight junction proteins when compared to the ethanol-saline treated mice. While we postulate this as a potential mechanism, future uptake studies with ^14^C-butyrate into the portal vein may be interesting. 

Decreased transport of butyrate into colonocytes would lead to less butyrate entry into the liver via the portal vein. This likely explains the reduced expression of SLC5A8 in the liver of ethanol-saline mice when compared to those supplemented with the synbiotic. Interestingly, we found that the expression of SLC5A8 was visibly greater around the portal vein in pair-fed and synbiotic-supplemented mice. As we found that ethanol-induced markers of inflammation and oxidative stress also localized around the portal vein in the ethanol-saline treated mice, this may link an anti-inflammatory and antioxidant protective role of butyrate in mice receiving synbiotic supplementation. Additionally, our prior studies showed that mice exposed to chronic-binge ethanol had more hepatic injury around the portal vein when compared to ethanol-exposed mice supplemented with tributyrin [10]. Recently butyrate-producing bacteria, butyrate, and propionate have been shown to beneficially modulate energy expenditure and high-fat diet induced non-alcoholic fatty liver disease [46,47]. These effects are related to alterations in endocrine function involving insulin sensitivity, glucagon-like peptide 1 induction, and signaling through SCFA G-protein coupled receptors. Our data found reduced hepatic steatosis induced by chronic-binge ethanol exposure in synbiotic treated animals. These effects could be attributed to increased butyrate and propionate transport into the hepatocyte in these mice when compared to the ethanol-saline treated animals. Further studies characterizing the transport and metabolic effects of this designer synbiotic during ethanol exposure would be interesting.

## 5. Conclusions

Here, we demonstrated that supplementation with a designer synbiotic influenced luminal SCFA and expression of SCFA transporters in the proximal colon and liver in mice, and these effects were associated with protection against chronic-binge ethanol-induced disruptions in colonic tight junction protein expression, hepatic inflammation, and oxidative stress as well as resultant hepatocyte injury and steatosis. These data suggest a targeted nutritional approach to counteract negative impacts on gut microbiota and its metabolites induced by ethanol exposure is a logical means to mitigate ethanol-induced gut-liver injury. Future studies investigating a role in human models are warranted.
